# Proceedings of the Canadian Society of Allergy and Clinical Immunology Annual Scientific Meeting 2018: Part Two

**DOI:** 10.1186/s13223-019-0350-5

**Published:** 2019-06-24

## Real world data of Canadian’s living with Hereditary Angioedema: Part 1—Demographics

### Jacquie Badiou^1^, Linda Howlett^1^, Anne Rowe^1^, Kim Steele^1^, Jenna Falbo^2^, Stephanie Santucci^2^, Jodi Valois^2^, William H. Yang^2,3^

#### ^1^HAE Canada, Ottawa, Ontario, Canada; ^2^Ottawa Allergy Research, Ottawa, Ontario, Canada; ^3^University of Ottawa Medical School, Ottawa, Ontario, Canada

##### **Correspondence:** Jacquie Badiou, Linda Howlett, Anne Rowe, Kim Steele, Jenna Falbo, Stephanie Santucci, Jodi Valois, William H. Yang

*Allergy Asthma Clin Immunol* 2019, **15(Suppl 3)**

**Background:** Hereditary angioedema (HAE) is an unpredictable and serious genetic disorder affecting approximately 1:10,000 to 1:50,000. It is an autosomal dominant disorder due to C1 inhibitor deficiency. Clinically, it is manifested by painful, unpredictable edema of the face, larynx, abdomen, genitals and extremities. It can be debilitating and if left untreated, may be fatal. We sought to better understand the demographic profiles of patients living with HAE in Canada.

**Methods:** In 2017, a comprehensive survey was sent out to all HAE Canada members by email to gather information on HAE in Canada. Data from respondents have been collected and analyzed using percentage of total surveys to provide data on demographics of these patients.

**Results:** The demographic location of HAE patients living in Canada includes Ontario, Alberta, Manitoba, British Columbia, Nova Scotia, Quebec, Saskatchewan and Newfoundland and Labrador. 140 respondents indicated their relationship to HAE as; 81% are adults living with HAE, 10% are caregivers of an adult living with HAE who lives with them, 2% are caregivers of an adult living with HAE who does not live with them, 2% are adults awaiting a diagnosis, and 4% are other or unknown. 109 respondents indicated 79% are female and 21% are male. When respondents were asked about their HAE type, 60% were found to have type 1/2 C1-inhibitor protein deficiency, 26% have HAE with normal C1-inhibitor, 10% unsure, and 4% have acquired angioedema.

**Conclusions:** This survey helps to better understand the current demographic profile of patients living with HAE and is the first national HAE survey done in Canada. However, data interpretation is limited due to uncertainty of necessary sample size required to be representative of the true population. Overall, our results demonstrate that HAE patients can be found across Canada and that the majority of patients in this survey are aware of their diagnosis.

## Real world data of Canadian’s living with Hereditary Angioedema: Part 2—Attack Profile

### Linda Howlett^1^, Jacquie Badiou^1^, Anne Rowe^1^, Jenna Falbo^2^, Stephanie Santucci^2^, Kim Steele^1^, Jodi Valois^2^, William H. Yang^2,3^

#### ^1^HAE Canada, Ottawa, Ontario, Canada; ^2^Ottawa Allergy Research, Ottawa, Ontario, Canada; ^3^University of Ottawa Medical School, Ottawa, Ontario, Canada

##### **Correspondence:** Linda Howlett, Jacquie Badiou, Anne Rowe, Jenna Falbo, Stephanie Santucci, Kim Steele, Jodi Valois, William H. Yang

*Allergy Asthma Clin Immunol* 2019, **15(Suppl 3)**

**Background:** Hereditary Angioedema (HAE) is a rare genetic disorder that is characterized by episodes of unpredictable painful swelling in different body parts involving the face, larynx, peripheral limbs, abdomen and genitals. In Canada, there are approximately 400–600 HAE subjects. To better understand the challenges of Canadians living with HAE we conducted the first web survey among our HAE Canada members, the objective was to gather real world data that will provide insight into the attack profiles of a HAE patient.

**Methods:** In 2017–2018, data was collected through voluntary online surveys of children, youth, and adults who live with HAE and their caregivers in Canada. The following data was based solely on adult participants.

**Results:** Among 104 participants with HAE they reported a diagnosis of: Type 1 or 2 C1-inhibitor protein deficiency (60%), HAE with normal C1-inhibitor (26%), acquired angioedema (4%), and unsure of diagnosis (10%). In the last year, 78% were symptomatic, 11% were asymptomatic, and 11% were unsure. Regarding the frequency of attacks: 61% had 7 or more attacks, 22% had 1–6 attacks, 6% had no attacks, and 10% were unsure. Identifiable attack triggers vary from stress (87%), typing/writing (78%), trauma (70%), illness (61%), medical procedures (61%), anxiety (55%), and Ace Inhibitors (6%). Other factors that increase HAE symptoms include menopause (9%), estrogen contraceptives (33%), and menstruation (47%). To treat these attacks, 84% use an agent, compared to 16% who do not. The most common treatment agent used was C1 esterase inhibitor (Berinert IV).

**Conclusions:** Our findings demonstrate the majority of participants are knowledgeable in identifying their triggers and managing their attacks. Results show improvements are necessary for proper diagnosis and awareness of the disease. Since the number of people living with HAE is estimated, our data is limited to the respondents and may not represent the broader Canadian HAE population.

